# Control of Foodborne Biological Hazards by Ionizing Radiations

**DOI:** 10.3390/foods9070878

**Published:** 2020-07-03

**Authors:** Muhammad Tanveer Munir, Michel Federighi

**Affiliations:** 1 LIMBHA, Ecole Supérieure du Bois, 7 rue Christian Pauc, 44000 Nantes, France; 2 Oniris, UMR 1014 SECALIM Oniris/INRAe, route de Gachet, CS 40706, CEDEX 3, 44307 Nantes, France

**Keywords:** ionization, radiation, food safety, microbiology, preservation

## Abstract

Ionization radiations are used to ensure food safety and quality. This irradiation process uses ions of beta or gamma rays to inactivate or destroy the food spoilage pests, microorganisms and their toxins without significantly increasing the temperature of the treated product. Meanwhile, various intrinsic and extrinsic factors are involved in determining the efficacy of ionization irradiation against these organisms. Therefore, the dose of radiations is recommended according to the type of irradiation, substrate and microorganisms. However, controversies are surrounding the use of irradiations in the food industry due to a negative perception of irradiations. This manuscript described the use of ionization radiations to control the foodborne biological hazards and increase shelf life. Firstly, the characteristics and mode of action of irradiations were discussed. Secondly, the role of extrinsic and intrinsic factors influencing the radioresistance of biological hazards were elaborated. This literature review also detailed the differential effects of irradiations on different microorganisms and pests having a role in food safety and deterioration. Finally, the regulatory status and the consumer values along with the controversies surrounding the use of ionization irradiations in the food sector were explained.

## 1. Introduction

**Figure 1:**
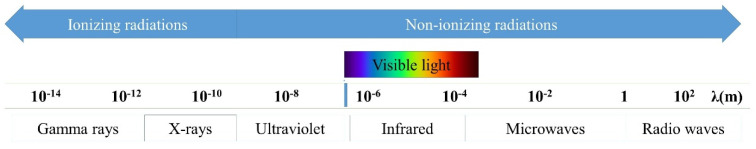
Electromagnetic spectrum.

Radiation is the energy that emits from one source to another in space or in a material environment [1]. Various forms of radiant energy are emitted from an electromagnetic radiation spectrum (Figure 1). The ionizing irradiation is the process of applying ionizing energy to a material, such as food, to sterilize or preserve them by destroying microorganisms, parasites, insects, and other pests [2]. Ionizing radiations can remove bound electrons from an atomic or molecular structure, leaving the structure to become electrically charged or ionized [1]. The major ionizing radiations used in food industries are: electromagnetic gamma (γ) rays obtained from radioactive forms of the elements cobalt (^60^Co) or cesium (^137^Cs), and the corpuscular beta rays (β) that are the electrically formed electrons in a cathodic tube, which are taken by an electron accelerator to form a stream of high-speed electrons propelled to foods [3,4].

The use of ionizing radiation technology, also named ionization or irradiation, to sterilize the single-use medical equipment started in the late 1960s. Since then, it has become a major application for laboratory material sterilization and improvement of material quality at very high doses ranging from 40 to 100 kGy [5,6]. At the same time, the Agro-Food Industries (AFI) became interested in this process, which seemed to have certain advantages, such as preserving food quality and higher preservation potential, as compared to conventional heat treatments. The ionizing radiation started being used on various foodstuffs to improve microbiological safety and/or extend the shelf life [7]. Meanwhile, the regulatory and scientific frameworks started building up in the following years. In particular, the effect of radiation on microbiological agents and the possible toxicity of radiation-induced molecules have been the subject of many studies [5,6,7,8].

This literature review aimed to summarize the important knowledge of ionizing radiation technologies to improve the shelf life and safety of food. It introduced the readers about the basic concepts of food ionization, their applications in food industries and mode of action against microbes, and regulatory status. This article would guide the consumers about the safety of these techniques, the trainers would find the basic knowledge necessary for their teaching, and food industry professionals would get information about the mode of application.

## 2. Methods of Production and Application of β and γ Rays

Although β and γ radiations have different origins, their mode of action is similar [9]. According to IAAE (International Agency for Atomic Energy), ionizing radiation for the treatment of foods can be safely applied with the specific condition of energy limitation of sources [10]. The energy of electron beams (E-beam) or beta rays (β) should not exceed 10 million electron-volts (MeV) [11], and the energy of γ rays installation should be less than 5 MeV [5].

The β rays are highly ionizing electrons, which means they have a mass and when they come in contact with matter they rapidly lose their energy. Therefore, they have low penetration power and their application is limited to homogeneous and low-density products [5]. On the other hand, the γ rays are the photons with no mass, and thus they are less ionizing and can penetrate deeper into the matter. As a theoretical example of a material with density one, the penetration of β rays is 3 cm while the penetration of γ rays for the same product would be 12 cm.

### 2.1. Electron Accelerators

Electron accelerators are electrical machines that make it possible to obtain a flow of electrons whose characteristics in terms of energy, intensity, and geometry are controlled [9]. The principles of acceleration are varied but the main components of these machines always remain the same: an electron source (cathode), an accelerator tube and a beam shaping system [12]. A cathode (made of tungsten, for example) is electrically excited in a vacuum system and will emit electrons that will be picked up and subjected to a potential difference in an electron gun [5,11]. The electrons are thus accelerated to an energy proportional to the voltage used, then shaped to form a beam that will be directed towards the product to be treated [4].

The beam current (*I*) is expressed in milliamperes (mA), which determines the dose rate and potential throughput. The delivered dose (*D*) is directly proportional to *I* and inversely proportional to the velocity of the product (*v*), making the relation of *D* = *k* (*I*/*v*). Whereas, the coefficient *k* varies with different factors including the distance between the product and the point of beam emergence, the scanning width and the geometry of the conveyor system. The power of accelerator is the product of *I* and electron energy [9]. For example, the power of a 10 MeV accelerator at 20 mA is 200 kW.

Generally, the products to be treated pass under the beam through a conveyor system adapted to the principles of radiation protection [9]. When the electrical excitation of the cathode ceases, it is obvious that the flow of electrons also ceases [12]. This on/off operation can allow the installation of this equipment in production plants, as the radiation emission is not permanent [9,13].

The application of E-beam technology also depends upon the energy of these radiations. The low energy (0.1–1 MeV) E-beam technology applications include surface sterilization, aseptic packaging and food packaging modifications. The medium energy (1–5 MeV) E-beams can be used for surface sterilization, food pasteurization in customized packaging, and packaging material modifications. While, the high energy (5–10 MeV) E-beam technology applications include the food sterilization and phytosanitary treatment, pasteurization, and waste treatment of food industry [13,14,15,16].

### 2.2. Radioactive Sources

The common radioactive sources are caesium-137 (^137^Cs) or cobalt-60 (^60^Co). Industrial facilities mainly use radiation generated by Co^60^ that is an artificial radionuclide produced in nuclear reactors from Cobalt 59, a stable non-radioactive metal. Radioactive ^60^Co emits radiation and two photons of energy 1.17 and 1.33 MeV [5]. The implementation of radiation treatments is based on the exposure of the product, for a given time, to the radiation emitted by the radioactive source. The ^137^Cs can also be used for this purpose, however, it is now only limited to laboratory facilities owing to radiation safety concerns and higher price of the element [9].

The ^60^Co used in industrial installations consists of rods that are stacked on each other and enclosed in the double stainless steel casing [9,17]. These sources are positioned in source holders of variable geometry (flat, cylindrical, square, etc.) depending on the type of installation [9]. The International System of Units (SI) recognizes Becquerel (Bq) as the unit of activity of these sources, and it is equal to the number of radioactive decays per second [5]. The other non-SI-unit, curie (Ci) is also used and it is the activity of one gram of ^226^Ra or 3.7 × 10^10^ Bq [9]. The continuous decay of radionuclides changes their activity over time. This decay time is represented by half-life, which means that at the end of this period, the activity is half of the initial activity [9]. The half-life of ^60^Co and ^137^Cs is 5.26 and 30.17 years, respectively [18].

The radioactive sources are immersed in a deep pool filled with water at about 8 m for storage. The products are exposed to rays via a conveyor system with discontinuous supply [9]. To ensure that the center of the product has received the desired dose all treatments can be done in the specialized multi-sided exposure facilities [5]. Gamma irradiators are designed to work on the fail-safe principle. In case of an undesired accident, the source returns to the safe storage position [11].

The overall activity of radioactive materials implemented in industrial installations varies from a few hundred thousand to a few million Ci [17], and at this level of activity, a radiation treatment facility is considered a Basic Nuclear Facility [5]. Therefore, the products to be treated are sent to these specific facilities contrary to electron accelerators, which can be installed in the food companies and products can be treated there.

## 3. Effects on Microorganisms and Pests

### 3.1. Mode of Action of β and γ Rays

Both the β and the γ rays consist of the kinetic energy of electrons and photons, and when it comes in contact with the target product, it leads to the ionization and excitation of matter in forms of physiochemical effects [18]. The rupture of covalent bonds involving ejected electrons leads to the formation of radiolysis products (free radicals). It is important to note that most of the free radicals formed have a very short life span and cannot be used as indicators of treatment [11]. Their quantity will depend on the absorbed radiation dose and the degree of hydration of the product. In some cases, such as dry or bone-in products, some free radicals will have a long life and will not recombine immediately after formation. Stable bone-induced products, such as 2-alkylcyclobutanones are one example of trace molecules from ionizing treatments of meat, chicken and eggs, which can be used as an indicator of irradiation [19]. The majority of radiation energy goes into the creation of hydrogen and hydroxyl radicals from water molecules and since the exposed foods are usually hydrated, the radiolysis of the water in food is responsible for the inactivation effects of microorganisms [5,18].

**Figure 2:**
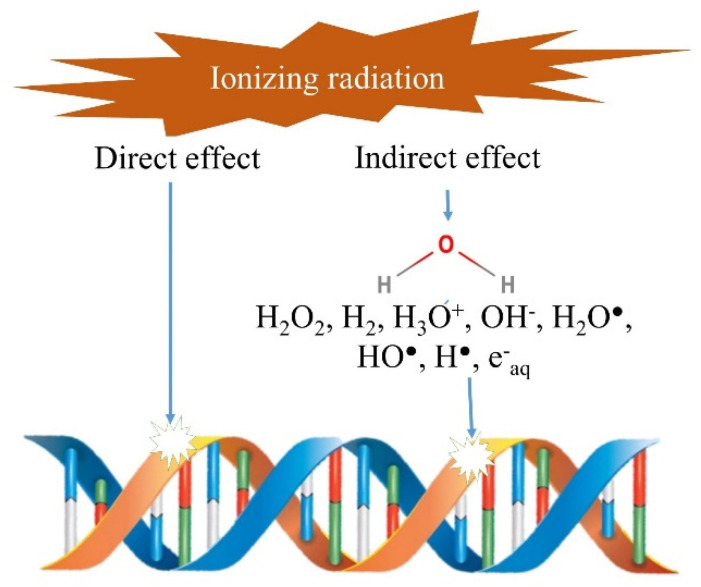
Schematic presentation of the effect of ionizing radiations on the nucleic acid. Left part, the radiations directly break the bonding between the base pairs in genetic material (DNA or RNA) causing the reproductive death of cells. Right part, the indirect damage to DNA (and other cellular components) is caused by the free radicals ad reactive oxygen species generated by the breakage of water molecules.

In living cells, e.g., yeasts, molds and bacteria, the irradiation effects through the induction of genomic, biochemical, physiological and morphogenetic changes [20]. Consequently, it has long been accepted that the mode of action of ionizing radiation consists of a more or less pronounced denaturation of the cellular material. One of the main effects is the direct energy dissipation and damage to macromolecules such as nucleotides by breaking single and double bonds and inducing the cell apoptosis (Figure 2, left). Furthermore, indirect radiochemical effects play role in cell inactivation, for example, the formed free radicals and reactive oxygen species can damage nucleic acids (Figure 2, right) and other cellular material or compartments e.g., membranes and key enzymes [5]. The hydroxyl OH^●^ radical is the most common disruptor due to its strong oxidizing power. This radical is responsible for irreversible damage to the biological molecules near its formation. Other highly reactive entities such as H_3_O^+^, H^●^ and e-hydrated (the hydrated electron) are also formed during the radiolysis of water and play an important role in cellular damage.

### 3.2. Doses of Irradiation for the Microbial Inactivation, Pest Sterilization and Inhibition of Germination

Irradiation dose is the amount of energy absorbed by the food while it is exposed to the irradiation field [18]. The unit for this absorbed dose is the Gray (Gy). The following equivalences are allowed: 1 Gy = 100 rads = 1 joule/kg. The dose rate, however, is measured in kGy/h for γ and kGy/s for β [9]. The inactivation of microorganisms by ionizing radiation is highly dependent on this dose.

**Figure 3:**
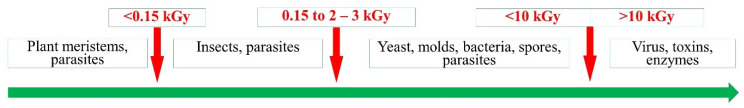
Range of Lethal Doses (LD) for different biological treatment targets (the LD for a human being is 10 Gy).

Small and simple DNA viruses are among the most resistant organisms to ionization (Figure 3). The next comes the bacterial or fungal spores due to their poorly hydrated cytoplasm, followed by vegetative cells of bacteria, molds and yeasts over a relatively wide dose range. To affect the vital functions of insects, the doses required will be lower. Parasites and plant meristems, for example, germs of bulbs and tubers, are more sensitive to ionizing radiation. At the end of the scale are humans with a much more complex DNA and a highly hydrated cell cytoplasm [18].

### 3.3. The Principle of Radiobacteriology

Ionizing radiation has lethal effects on micro-organisms. Their destruction will meet certain general conditions that will have to be modulated according to various factors of extrinsic, intrinsic, protective or favorable variations. The general conditions obey a mathematical law governed by the equation:

$$N=No\cdot e^{kdose}$$

This equation [21] reminds us of the one more known in thermobacteriology,

$$N=N_{o}\cdot e^{kt}\;(\mathrm{or}\;N=N_{o}\cdot e^{-t/D})$$

This equation shows that there is an equivalence between the time/temperature concept specific to heat treatments and the notion of absorbed dose for radiation. Here *k* is the constant of microbial destruction. *N* is the number of surviving cells at time *t*. *D* is the decimal reduction time. This equation thus depicts:

- That the number of microorganisms destroyed depends on the initial number (*N_o_*).
- That for each equivalent dose of ionizing radiation, there is an identical proportion of microbial population destroyed (cumulative effect).
- That according to the exponential law we cannot reach zero.

The Decimal Reduction Dose (DRD also called D_10_) is the dose of ionizing radiation (expressed in Gray) required for a 90% inactivation of viable Colony Forming Unit (CFU) or by one logarithmic cycle [22]. However, many factors influence these values, and they need to be known to better control the treatment and its result on microbial populations [7,11].

### 3.4. Factors Affecting the Kinetics of Destruction of Microorganisms by Ionizing Radiation

#### 3.4.1. Intrinsic Factors

The intrinsic factors influencing the D_10_ values are related to the nature of microorganism itself. They include the type, strain, growth stage, fitness and stress level of microorganisms. In general, bacteria are more radiosensitive than viruses, for example, as seen in Figure 3. It is common to say that radio-sensitivity is inversely proportional to the degree of organization/complexity of the organism (Lethal Dose for humans is 10 Gy versus D_10_ of viruses, which is >10 kGy).

The cell wall structure has a role in bacterial radioresistance. The Gram-negative bacteria are more sensitive to radiations as compared to Gram-positive bacteria. Likewise, the aerobic bacteria have lower radiotolerance as compared to anaerobic counterparts [23]. Radiosensitivities generally varies with D_10_ of about 0.01 to 1 kGy for vegetative forms of bacteria [11]. However, some bacteria are non-spore-forming, such as *Deinococcus radiodurans, Deinococcus radiophilus* and *Moraxella sp.*, which have the particularity of being highly resistant to ionization (D_10_ values of 2 to 8 kGy) [5,11,24]. The earlier identified reason for this resistance was that these species possessed a particularly efficient system for the repair of radio-induced DNA lesions [25]. Later, it was identified that the ratio of intracellular Mn/Fe concentrations plays a role in radioresistance in these bacteria [24]. The model studies have been performed on *Shewanella odeinensis* (iron-rich, manganese-poor) radiosensitive (DRD 70 Gy) and *Deinococcus radiodurans* (manganese-rich, iron-poor) in which 10% of the population can withstand 12,000 Gy. The strong correlation between radioresistance and a high Mn/Fe ratio has been found (Table 1) [26,27]. According to Ghosal et al. (2005), the manganese ions (Mn^2+^) accumulated in bacteria served as antioxidants, reinforcing the enzymatic defense systems against oxidative stress [27].

The D_10_ values of most common foodborne pathogens are between 0.4 to 0.7 kGy [22,28]. Sommers and Boyed [29] reported that the mean D_10_ values (kGy) for *Escherichia coli* O157:H7, *Salmonella*, *Listeria monocytogenes* and Staphylococcus aureus were 0.45, 0.65, 0.55 and 0.58, respectively. The radiosensitivity can be different in different strains and isolates of the same species of bacteria [30,31]. Xu et al. [31] studied the radioresistance of 25 individual *E.coli* isolates inoculated on the ground chicken meat. The D_10_ values in isolates of clinical uropathogenic *E. coli*, newborn meningitis causing *E. coli*, isolates from retail chicken meat, as well as retail chicken-skin isolates, were 0.25, 0.29, 0.29, and 0.39 kGy, respectively. The mean D_10_ value for the clinical isolates was 0.27 kGy vs. 0.34 kGy for the non-clinical isolates.

The D_10_ values are also influenced by the presence of different competitive microorganisms in the same medium. Competitive stress reduces the radioresistance of microorganisms as shown in Table 2 [32].

The radioresistance of microorganisms may increase when they adapt to the stress conditions [33]. The sublethal doses of irradiation itself could be a culprit. Levanduski and Jaczynski [34] reported that the D_10_ values of *E. coli* in ground beef increased (*p* < 0.05) with subsequent cycles of E-beam processing, starting at 0.24 kGy for *E. coli* ATCC strain 35,150 and reaching 0.63 kGy for *E. coli* isolate L3. Following four cycles of E-beam processing, the isolate L3 increased (*p* < 0.05) its radio-resistance and survived an E-beam dose of 3.0 kGy. The exact mechanism for this radioresistance is unknown. Initially, it was postulated that the DNA repairing ability of microbes helped them to become resistance [34]. However, it was later observed that this resistance to E-beam also developed in those strains of *E. coli*, which lacked the genetic repairing ability [35]. Sometimes, pathogens may acquire certain resistance genes to some specific antibiotics and they may also help them to tolerate high doses of irradiation. For example, Gaougaou et al. [33] observed that in the *E. coli* O157:H7, γ irradiation at 0.4 kGy dose increased ampC and ampG expression, respectively, by 1.6 and 2-fold in the wild type strain but up to by 2.4 and 3.4-fold when the strain was beforehand adapted to 25 μg/mL of carbenicillin. Likewise, Skowron et al. [36] observed that the antibiotic-resistant *L. monocytogenes* strains were more resistant to both the β and γ radiations. However, it was observed that the E-beam treatments did not increase or decrease in the expression of virulence genes of *L. monocytogenes* during the storage time of sliced dry-cured ham [37]. Further research is required on this subject because such considerations are important for the food industry, especially when considering the use of ionization radiations for the preservation of meat obtained directly from animals fed antibiotics [33].

In general, bacterial spores are more radioresistant than vegetative forms [25]. *Clostridium botulinum* in the form of spores is the most radioresistant (DRD of 1 to 3.3 kGy) and microbiologically important food hazard [38]. The other important spore-forming bacteria are *Bacillus cereus* and *Clostridium perfringens* [5,28].

It should also be noted that there is no direct association between resistance to heat and resistance to ionizing radiation. For example, *Moraxella sp*. is very radioresistant and is easily destroyed by heat, whereas *Bacillus stearothermophilus*, which is very resistant to heat, is much more sensitive to ionizing radiation [5]. Therefore, combinations of heat-ionization treatment can be considered in some cases [28].

The radioresistance of bacteria also depends upon the growth phase. It has been reported that the cells in the exponential growth phase are more sensitive to ionizing radiation than for microbial populations in the latent or stationary phase [5]. However, the studies on radiation resistance of *D. radiodurans* concerning different phases of growth have shown that late stationary phase cells are four-fold more sensitive to irradiation and heat as compared with exponential or early stationary phase cells [39].

However, it has also been shown that preparatory stresses such as nutritional deprivation (starved cells), can lead to an increase in the DRD of a strain, all other things being equal. Thus, Mendonca et al. [40] compared DRD values of *Listeria monocytogenes* scottA in exponential phase, stationary phase and after 12-day stress in saline solution. They showed that the DRD for stressed cells was significantly higher (0.21 kGy) than that of cells in the stationary phase (0.09 kGy), which was itself higher than that of cells in the exponential phase (0.07 kGy) [40]. Similarly, Buchanan et al. [41] demonstrated a 1.2 to 3.3-fold increase in DRDs in *E. coli* O157:H7 from seven strains that had been pre-adapted to acid stress (several organic acids tested). However, this effect may vary in different types of microorganisms. For example, Osaili et al. [42] reported that there was no significant difference in D_10_ values in starvation and heat stressed *Salmonella*.

Intrinsic factors alone cannot explain the differences in radioresistance in different microorganisms. Therefore, the knowledge of the effects of extrinsic factors is necessary to establish the treatment of radiation values. In this regard, previous research on *Cronobacter sakazakii* (old name *Enterobacter sakazakii* [43]) in the 2000s, is a very good example. These microorganisms have caused major problems in powdered milk preparations and have been controlled by ionizing treatments [44,45]. According to Lee et al. [44], the DRD value of *E. sakazakii* in dry infant formula was 0.76 kGy, whereas it was 1.71 kGy in dehydrated infant formula for Osaili et al. [45]. Later, Hong et al. [46] reported a DRD value for *E. Sakazakii* of 4.83 kGy for dehydrated weaning food rich in rice flour. They explained this strong difference by the very different formulation of their product as well as its composition that was very rich in antioxidants, especially manganese, and the treatment by accelerated electrons contrary to the two other studies carried out by gamma radiation [46].

#### 3.4.2. Extrinsic Factors

Influence of processing and culturing media: In all types of physical preservative treatments, it is quite common to find a difference between the food matrices. This is most often associated with the protective effect of food [47]. Ionizing treatments are no exception to this rule, the nature of the treatment environment, whether by its composition or physical state, affects the dose required to kill a Log, compared to that required to achieve the same result in vitro (Table 3). Kawasaki et al. [48] reported that the D_10_ value of *E. coli* and *S.* Enteritidis in the beef liver was higher than that of ground beef under different irradiation conditions.

The choice of the cultivation method and the timing of its use for the enumeration of microorganisms post-treatment are also very important [5,50]. Indeed, in general, the use of a selective enumeration medium rich in antibiotics, for example, gives lower D_10_ values than in semi-selective or non-selective media (Table 4). In an overly selective medium, repair mechanisms would be inhibited and/or delayed, resulting in a slower recovery of bacteria stressed by ionization and thus lower bacterial counts in this medium. This reasoning also applies to any other stress (heat, cold, acids) suffered by bacteria [49,51].

Influence of temperature and free water content of the treatment medium: It has been described that it is possible to combine heat treatments (at lethal temperature for microorganisms) and ionizing treatments to amplify the sterilization effects. The idea behind this combination is to perform these treatments at sub-optimal levels to limit the harmful effects on the organoleptic qualities and physical properties of the product.

At different temperatures, the D_10_ values of same products can be different, as Zhang et al. [30] observed that the D_10_ values (kGy) of low beam ionization radiation of *Bacillus subtilis* spores at 80 keV and 200 keV were 3.0 and 2.5 for 37 °C while both were 2.2 at 30 °C. At higher temperature differences, such as room temperature and below zero, the differences of D_10_ values would also be greater [48].

In general, it is known that microorganisms are more resistant in a frozen medium than in a medium at room temperature, or in a dry medium than in a water-rich medium (Table 5). Skowron et al. [36] reported that for the *L. monocytogenes* strains the lower the temperature at which the contaminated fillet fragments were exposed to radiation, the higher the theoretical lethal and D_10_ doses were, irrespective of the type of radiation.

This effect of water is also found in low water activity environments, where a higher dose of ionizing radiation is required to achieve the same effects, compared to a high water activity environment [49].

Another example of temperature and ionization effect combination in the food industry is the deworming of meats, where anti-parasitic effects of freezing and ionizing radiation can be used together [5]. It should also be noted that the treatment of frozen products is recommended for high doses, as the adverse effects of treatment (appearance of unpleasant flavors for certain categories of products and changes in texture and color) are less pronounced than in the case of treatment at room temperature [52].

Influence of the atmosphere during ionization treatment: The presence of oxygen at the time of treatment would be known to potentiate the lethal effect of ionization [5,53,54] (Table 6). Moreira et al. [55] reported that *E. coli* and *Salmonella* Typhimurium were more resistant to ionizing radiation when irradiated under vacuum than at atmospheric conditions. The D_10_ value of both bacteria in all produced commodities increased more than two-fold (*p* < 0.05) compared to the irradiated controls (sealed bags without vacuum).

However, this is not always verified, a vacuum treatment can lead to lower D_10_ values than an air treatment, probably due to the accumulation of the stresses to which the microorganism is subjected [56]. Sommers and Boyd [28] reported that the modified atmosphere with 100% N_2_, 50% N_2_ plus 50% CO_2_, or 100% CO_2_ did not affect the radiation resistance of *E. coli* O157:H7, *Salmonella*, *L. monocytogenes* and *S. aureus.* Hume et al. [50] also reported that there was no effect of air volumes on the radioresistance of RNA viruses. Here, no effect of air or vacuum can be attributed to the direct effects of irradiations. However, further research in this field can help to identify the optimal atmospheric conditions along with irradiation to achieve higher food safety goals [14].

The variations due to packaging material: Commonly used food packaging materials including polyethylene (60%), polypropylene (15%) and polystyrene (5%) are generally compatible with irradiation [57]. However, the microbial resistance to ionization treatment can also be influenced by the packaging material used for food products [58]. Kortei et al. [59] reported that the mean DRD values (kGy) for *Bacillus cereus* in polypropylene packaging for fresh and dried mushrooms were 3.21 and 2.40, respectively, and for polythene, they were 0.76 and 1.80 respectively. To avoid the influence of packaging materials on radioresistance of microbes in food, they are commonly irradiated prior to filling in the dairy (butter, cream, eggnog), processed food (sauces, fruit gels, processed meats, salad dressings) and beverage (wine, juice) industries [60]. It is done especially for E-beam because of their low penetration as compared to gamma radiations, which can be effectively employed even with thicker packaging materials.

Effect of the energy level of ionization radiation: The type of ionization and their energy level can influence the radioresistance of microorganisms. Zhang et al. [30] reported that there were variations in D_10_ values of spores from *Geobacillus* species with an E-beam at energy levels of 80 and 200 keV, however, these variations were in the range of 2.2–3.0 kGy, and 2.2–3.1 kGy, respectively. It is also important to note that the difference in D_10_ may also depend upon the origin and type of irradiation [36].

Influence of additives during ionization treatment: The safety of foods is also obtained by the hurdle technology approach. Ionizing radiation can be combined with several physical or chemical preservation treatments [29,50,61,62]. Dutra et al. [38] combined ionization treatment and different levels of nitrites to inactivate *C. botulinum* spores in mortadella. High doses (>10 kGy) of radiation were required for the inactivation of *C. botulinum* spores in the absence of nitrites. With 150 ppm of nitrites in mortadella, the same anti-botulinic effect needed a lower dose of radiation which was better for the organoleptic properties of mortadella [38]. In this study, the nitrites worked as a hurdle with its anti-*Clostridium botulinum* activity, and the weakening of pathogen made ionizing radiations more effective.

### 3.5. Effect on Bacteria

There is a great diversity in the behavior of bacteria to ionizing radiation. In addition, D_10_ values obtained from the literature should be used with caution even though they provide valuable information on the expected sensitivity of the microorganism(s) to ionization for respective applications (Table 7 and Table 8).

In addition to this presented information, it should be noted that bacterial toxins are highly radioresistant, with botulinic toxin having DRD values between 17 and 60 kGy and staphylococcal toxin between 27 and 95 kGy [38,63,64]. It is, therefore, obvious that when the toxin is already present in the food, usual ionizing treatments cannot be effective for its elimination.

**Table 7:** DRD values (in kGy) of the main foodborne bacterial hazards.

Bacteria	DRD (kGy)	Treatment Media	Temperature
*Aeromonas hydrophila*	0.14–0.19	Minced beef	+2 °C
*Bacillus cereus* (spores)	3.2	Nutrient broth	Room
*Bacillus cereus* (spores)	2	Water	Room
*Brucella abortus*	0.34	Minced beef	Room
*Campylobacter jejuni*	0.16	Minced beef	+0–5 °C
*C. botulinum type A* (spores)	3.3	Phosphate buffer	Room
*C. botulinum type B* (spores)	3.3	Phosphate buffer	Room
*C. type E* (spores)	1.4	Irish beef	Room
*C. perfringens type C (spores)*	2.1	Water	Room
*Cronobacter spp.*	1.71	Milk powder	Room
*Cronobacter spp.*	0.76	Milk powder	Room
*Cronobacter sakazakii*	4.83	Dehydrated milk	Room
*E. coli* O157:H7	0.27	Chicken meat	+5 °C
*Listeria monocytogenes*	0.42–0.49	Chicken meat	+10 °C
*Listeria monocytogenes*	0.21	Endive	+2 °C
*Listeria monocytogenes*	0.5	Broccoli	−5 °C
*Listeria monocytogenes*	0.76	Broccoli	−20 °C
*Salmonella* Enteritidis	0.33	Whole egg	+15 °C
*Salmonella* Newport	0.32	Whole egg	0 °C
*Salmonella* Panama	0.46	Chicken meat	+22 °C
*Salmonella* Paratyphi B	0.3	Crab meat	NA
*Salmonella* Typhimurium	0.4–0.48	Chicken meat	+22 °C
*Salmonella* Typhimurium	0.5–0.55	Whole egg	0 °C
*S.* Typhimurium	0.45	Ham	+10 °C
*Shigella dysentariae*	0.22	Shrimps	−18 °C
*Shigella flexneri*	0.41	Shrimps	−18 °C
*Staphylococcus aureus*	0.58	Minced beef	Room
*Staphylococcus aureus*	0.39	Ham	+10 °C
*Vibrio parahaemolyticus*	0.03–0.06	Fish	Room
*Yersinia enterocolitica*	0.1–0.21	Minced beef	Room

Adapted from [49,52,65]; NA = Unknown.

In terms of microbiological food safety, it is considered that the resistance to ionizing radiation of spores of *C. botulinum* is the reference in terms of radiation sterilization [5,38]. Thus, using the concept of commercial sterility (or concept of 12D), the minimum dose to obtain any type of radappertized food is 45 kGy [7,21]. In general, Table 7 shows that the main bacterial hazards in vegetative form do not have high radioresistance. It is generally the same for the bacterial agents of spoilage except for lactobacilli, which, for non-spore-forming bacteria, are relatively resistant to radiation (Table 8).

**Table 8:** DRD values (in kGy) of bacterial food alteration agents.

Bacteria	DRD (kGy)	Treatment Media	Temperature
*B. pumilus* (spores)	1.35	Nutrient broth	Room
*B. pumilus* (spores)	3	Dry medium	Room
*B. subtilis* (spores)	0.64	Saline water	Room
*B.stearothermophilus* (spores)	1	Phosphate buffer	Room
*Lactobacillus spp*	0.3–0.88	Minced beef	Room
*Lactobacillus spp*	0.55–0.81	Nutrient broth	Room
*Leuconostoc mesenteroïdes*	0.12–0.14	Water	Room
*Moraxella sp.*	4.7	Nutrient broth	Room
*Pseudomonas fluorescens*	0.12	Minced beef	Room
*P. putida*	0.08	Chicken meat	+10 °C
*P. aeruginosa*	0.07	Nutrient broth	NA
*Proteus vulgaris*	0.2	Oyster	+5 °C

Adapted from Desmonts et al. [49], NA = Unknown.

### 3.6. Effect on Yeast and Molds

Yeasts and molds are frequently present in food and their development will most often cause various food alterations (changes in texture, color, flavor and appearance). However, some molds, such as *Aspergillus flavus* and *Penicillium viridicatum*, are likely to produce mycotoxins, which are dangerous for humans and animals [66,67,68,69]. The sensitivity to ionizing radiation of yeasts and molds is generally of the same order as that of non-spore-forming bacteria [70]. The same parameters are likely to affect this sensitivity. The real lack of reliability of enumerating yeasts and molds, has led researchers to define a concept different from the DRD value: it is the dose of radiation that can prevent the growth of a known initial population (Table 9 and Table 10); however, some DRD values are also found in the literature [47,49].

Ionization irradiation can also be used to breakdown fungal toxins [71,72,73]. Domijan et al. [74] reported that the cellular toxicity and quantity of aflatoxin B1 and ochratoxin A (in methanol solution) were reduced by 5 to 10 kGy gamma irradiation. Earlier, Kottapalli et al. [69] also reported that the electron beam irradiation of *Fusarium* infected barley improved the quality of malt by reducing the infection and the deoxynivalenol toxin. Contrarily, sometimes, the sublethal irradiation doses can trigger the growth of some fungi and even the production of mycotoxins [75,76]. Therefore, the combination of treatments such as hot water (50 °C) washing of food products, along with ionizing irradiations is recommended to efficiently inactivate the mycotoxins [5,77].

### 3.7. Effect on Viruses (and Prions)

Only a few studies on viral inactivation by ionizing radiation have been conducted on foods [78,79,80,81,82]. The first reason for this problem is linked to the technical difficulties in carrying out these studies, and secondly, viruses are considered rather resistant to ionization [83]. The viral elimination by the sole action of irradiation treatment is possible only at doses that are generally outside the safe ranges used for foods (Figure 3). In the case of treatments in the agri-food sector (0.1 to 10 kGy, roughly), and if the objective is the elimination of viruses, it will be necessary to combine this with heat treatment, which is much more virucidal than ionizing radiation. Table 11 shows the DRD values for various viruses; here, the values are lower than 10 kGy which might be due to an additive effect of freezing. In general, there is an inverse correlation between viral genome size and susceptibility to inactivation by gamma irradiation [82,84], although there are exceptions that do not follow this trend [50].

The prions are more resistant to radiation treatment. The concept of D37 was developed, which corresponds to the dose necessary to reduce infectivity by 37%. However, in general, inactivation of prion deposits on materials is considered as impossible by using gamma irradiation alone [85]. Almost 50 kGy is required for an estimated 1.5 Log reduction of the scrapie agent in sheep [80].

### 3.8. Effect on Parasites

Parasites are biological hazards found primarily, but not exclusively, in the food of animal origin or water [87]. For example, *Toxoplasma gondii* can parasitize meat and indirectly water and raw vegetables (fecal contamination). It is especially dangerous for newborns contaminated by their mothers during pregnancy, leading to death, mental deficiency or eye disorders. *Entamoebea histolytica* can cause dysentery in humans through the consumption of water, raw fruits and vegetables contaminated with fecal matter. For these 2 types of protozoa, a dose of 250 Gy is considered sufficient to eliminate them from food. Lower doses may not be sufficient for the inactivation of parasites. For example, Pohle et al. [88] observed that very low doses between 50 and 100 Gy were insufficient to inactivate metacestodes of *Echinococcus multilocularis*. Other studies, discussing other parasites, reported very high doses for complete inactivation. This was notably the case for Yu and Park [89] who treated *Cryptosporidium parvum* oocysts with doses from 1 to 50 kGy and then passed them on a mouse model to test their viability. The mouse model allowed regeneration of *C. parvum* oocysts in all cases except those treated at 50 kGy [89]. Some examples of inactivation are shown in Table 12.

The effectiveness of ionizing radiation is highly dependent on the stage of development of a parasite, as shown in Table 12. Very high doses are required for the complete destruction of parasites. However, if the aim is to interrupt or disrupt the parasite cycle, lower doses will be sufficient. For instance, about 0.012 kGy dose is needed to sterilize *Trichinella spiralis*, while 0.020 to 0.030 kGy is needed for inhibition of their maturation and 1.4 to 6.3 kGy for their destruction.

### 3.9. Effect on Insects

The ionization doses required to kill an insect depend on its age at the stage of development. Thus, sensitivity to ionizing radiation varies greatly at different metamorphic stages of the egg, larva, pupa or adult. Again, as with the cell, this sensitivity increases with reproductive activity and decreases with the degree of differentiation. For example, the sensitivity of the eggs of the wheat yellow worm (*Tenebrio molitor*) is 250 times greater at 0.5 days of age than at 7.5 days of age. In practice, it is usually not necessary to achieve 100% lethality [49]. It may be sufficient to prevent the transformation of eggs and larvae into adults or to sterilize pupae or adult populations present. It should be noted that the ability to sterilize insects may be of great importance in quarantine treatments or biological control by releasing sterilized male insects into the wild. In general, females are more radiosensitive than males (see Table 13). Moths (Lepidoptera) are more resistant than mites, which, in turn, are more resistant than flies (Coleoptera). A dose of 250 Gy has been suggested as an effective quarantine treatment against fruit flies. It would prevent the emergence of viable adults from eggs and larvae [27].

## 4. Regulatory and Enforcement Status of Application of Ionizing Irradiation

### 4.1. Regulatory Aspects

The ionization of food products is a process subject to regulatory control. Authorized products are either part of an approved list or the authorization has been given by the competent authority based on an application for authorization. The implementation of treatments requires sophisticated installations and/or equipment in a perfectly controlled environment [9]. Therefore, ionization treatment is not as simple and globally developed as heat treatments. Several texts provide a framework for their use. At the global level, the texts of the Codex Alimentarius (2003a and 2003b) are references, they concern the general code of use 2003a and the practices of use 2003b and have been adopted by more than 60 countries [7,92,93]. This general framework is often supplemented by national and/or community regulations; for example, European directive 1999/3/EC provides a list of authorized products and their respective radiation doses [94].

**Figure 4:**
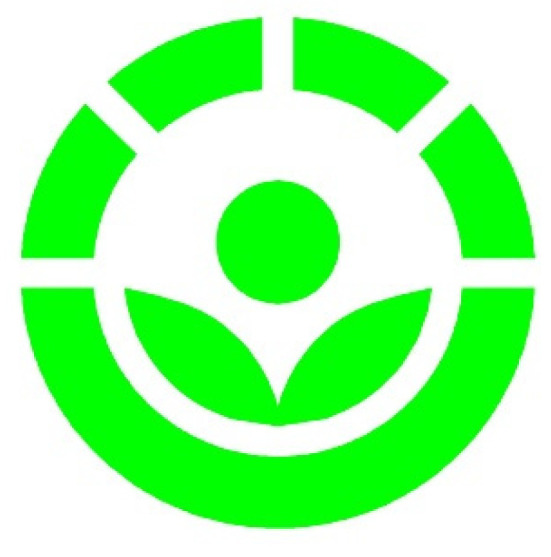
Radura symbol.

Consumer information on food treatments is extremely important, especially for treatments that are not always universally accepted. For ionizing radiation, information concerning its application to the food and/or one of the ingredients of the food is mandatory. This may be in the form of label statements such as “product treated by ionizing radiation”, “product treated by ionizing energy”, “product treated by irradiation” or the affixing of the international symbol “radura”, which indicates the existence of such treatments (Figure 4). However, the use of the radura symbol is voluntary [9,95].

### 4.2. Applications of Authorized Ionizing Treatments

There are several ways to describe the agri-food applications of ionizing treatments; the most common is based on the dose applied [96]. Different levels of ionizing radiations are authorized for different food materials (Table 14).

The terms radicidation and radurization are used to refer to these applications of less than 10 kGy doses [23,97]. Radicidation (from Latin: *radiare*—radiate and *ocsidere*—kill) is the application to the food of a dose of ionization sufficient to reduce the specific number of viable pathogenic bacteria to a level such that they are not detectable by any known method. This term also applies to the destruction of specific parasites. Radurization (from Latin: *radiare*—radiate and *durare*—prolong) is the application of an ionization dose sufficient to preserve the quality of food by ensuring a substantial reduction in the number of spoilage bacteria.

Radappertization (by the name of Nicolas Appert, the inventor of thermal sterilization method of food products) is the application of high dose (10 to 60 kGy) of ionization to food in order to reduce the number and/or activity of living microorganisms so that none (except viruses) is detectable by any recognized method [23]. Such radio-sanitized products can then be stored for up to 2 years at room temperature in sealed plastic packaging [5]. It should nevertheless be noted that the search for commercial sterility or microbiological stability remains a very marginal application of ionization (Table 15).

## 5. Controversies Concerning Consumer Perception and Acceptance of Food Ionization

Food irradiation has always been a divisive issue, in contrast to the widely accepted ionizing treatment of medical and laboratory equipment. The controversy regarding the application of ionization on food arises from the perception that the treated food is a living product and its consumption makes it part of the consumer’s body [98]. Besides, AFI has to face a big challenge in the use of ionization—harmonization of regulations and labeling all around the world- because it is a focal point for opposition to irradiated foods [99].

The microbicidal effect of ionization irradiation is widely accepted. However, the main limitations of the treatment are the high resistance of toxins and viruses, increased resistance in dry environments, etc. Proponents of the process play on this effectiveness and refer to the technical necessity of such treatments for various reasons. The first reason is to inactivate many pathogens and making food safe for consumption. The other reason is extending the shelf life of raw materials to avoid wasting food resources. On the other hand, the opponents of the ionization process repeat more or less the same arguments with a different point of view: (i) increasing the shelf life of products is a deception on the age and freshness of products and (ii) there is no “technical necessity” to increase the microbiological safety of products if all current procedures to ensure compliance with hygiene rules are implemented throughout the human food chain.

The safety of radioactive sources in terms of handling and the toxicity of stable, radiation-induced molecules, particularly 2-alkylcyclobutanones, is also debated. Song et al. indicated that toxic effects had been demonstrated in in vitro tests but toxicity to the consumer from the ingestion of food treated with ionizing radiation is very unlikely. They also stated that possible chronic toxicity remains to be evaluated [48]. Most often these are mutagenesis tests on bacterial strains in Petri dishes (Ames tests) which are only interested in the genotoxic effect, other authors indicating that it is the carcinogenesis-promoting effect of 2-alkylcyclobutanones that poses a real problem [100]. Finally, this subject is controversial, and the debate must remain open [101].

The ionization of food products enables the industry to develop and offer new products that meet changing consumer preferences and demands. The demand for ready to eat food is ever increasing due to the changing lifestyle and eating habits of the people. People are more aware of food safety and want to eat healthy and clean [57]. They almost always value fresh and natural foodstuffs [102]. Additionally, the recent food crises and uncertainty regarding food quality also have pushed consumers towards a growing need to know more about the foods they purchase, including information related to both food quality and production [103]. Therefore, the current developments in consumer demands and preferences can be met with the application of food ionization to increase the shelf life and safety of food products present in the market with uncompromised nutritional and sensory qualities [104].

The consumer interest depends upon various factors related to population characteristics. For example, a study in the Italian population showed that 89.2% of consumers were interested in receiving information on the treatment of foods with ionizing radiation aimed at raising product safety. Especially, this interest was higher in some specific groups, such as those who have a higher level of education, who reported a high sensitivity to food safety issues, and who had already or unwittingly purchased irradiated foods [104].

The studies have shown that the awareness and acceptance of food irradiation are influenced by multiple factors including the provision of proper scientific information [105]. A study in the Italian population showed that the acceptability of irradiated foods was mainly affected by the consumers’ perceived risk to health consequent to their consumption [103]. The socio-economic characteristics, such as age, monthly income and geographical area in which consumers live, also influence the choice [103].

In light of the literature findings, the following recommendations can be made to encourage consumers to accept gamma irradiation [102,103,104,105].

- The first step is communicating scientific information in public [105]. It can be done at different platforms, especially targeting consumer values. Galati et al. [103] emphasized the importance of specific food education programs and promotional campaigns supported by the ministry of education and other public and private institutions, aimed at creating greater awareness and attitude among consumers about irradiated products.
- The policymakers and food chain managers should also be involved in the promotion campaigns aimed to familiarize the consumers about the principles, aims and benefits of irradiation technology.
- The values of consumers should be addressed rather than the product. For example, highlighting the advantages of technology in keeping food “fresh” and “natural” would be more effective rather than just pointing out the technology.
- The information in promotional campaigns should take into account the positive and negative aspects of technology that will coexist in any food debate.
- Labeling the products to show advantage information as an assurance of food safety and customer values can decrease the consumer opposition to irradiated food.
- Create a partnership with food small or medium-sized retailers, so they can promote the marketing of irradiated food.
- Finally, take the stakeholders on the board who believe in the value of food irradiation, and thus food retailers will be seen as less biased and consumer trust will increase.

## 6. Conclusions

Ionizing treatments of food has been approved in more than 60 countries. The international and national bodies define a maximum dose limit for each approved food product.

Regarding the difference in the use of gamma radiations and E-beam technology, the gamma radiations have very high penetrability compared to E-beams. The cost of electron accelerators is very high and decreasing as the technology is being more common [13]. Meanwhile, the E-beams could be preferred because of having on/off production, and it allows their in situ installation. On the other hand, for gamma rays, it involves handling of radioactive material; therefore, the products are expected to be taken to treatment facilities [11].

The microbial toxins and viruses are considerably resistant to ionization irradiations. However, the combination of heat and ionization radiations can help to achieve food safety goals. Therefore, further research is needed to develop the guidelines and recommendations regarding combination treatments against biological hazards in food.

In recent times, the attitude of the general public towards this technology is positively changing, thanks to information transparency and awareness campaigns. Further, collaboration and partnership among different stakeholders can help to increase consumer awareness regarding the safety of these treatments.

## Figures and Tables

**Table 1:** Relationships between cytochrome-c number, Mn/Fe ratio and radioresistance of different bacterial strains.

Species	Genome Size (Mb)	Number of Cytochrome C	Intracellular Mn/Fe Ratio	DRD (kGy)
*Deinococcus radiodurans*	3.28	7	0.24	10–12
*D. geothermalis*	3.23	7	0.46	10
*Escherichia coli*	4.64	6	0.007	0.7
*Pseudomonas putida*	6.18	17	<0.001	0.25
*Shewanella odeinensis*	5.13	39	<0.001	0.07

Adapted from Daly et al. [26]; DRD: Decimal Reduction Dose.

**Table 2:** DRD values (kGy) of different bacteria in single and mixed cultures inoculated into wakye substrate.

Species	Single Culture	Mix Culture
*Escherichia coli*	0.27	0.24
*Staphylococcus aureus*	0.33	0.28
*Salmonella* Paratyphi B	0.44	0.32

Adapted from Adu-Gyamfi et al. [32].

**Table 3:** DRD values (in kGy) of *Salmonella* Typhimurium obtained in different food matrices.

Medium	Bone Marrow	Corned Beef	Whole Egg	Meat	Kaolin Clay	Phosphate Buffer
DRD	0.91	0.8	0.632	0.558	0.21	0.208

Adapted from Desmonts et al. [49].

**Table 4:** DRD values (in kGy) of *Listeria monocytogenes* obtained in different treatment and enumeration media.

	Treatment Media	
Numeration Media	Phosphate Buffer	Chicken Meat
*Listeria* selective agar	0.324	0.424
Tryptone soya levure agar	0.404	0.481

Adapted from Desmonts et al. [49].

**Table 5:** DRD values (in kGy) of *Salmonella* Typhimurium for different treatment media and temperatures.

	DRD Values (kGy)	
Treatment Media	At Room Temperature	At Frozen Temperature (−15 °C)
Phosphate buffer	0.208	0.391
Meat	0.558	0.963
Dehydrated coconut	1.58	-

Adapted from Desmonts et al. [49].

**Table 6:** DRD values (in kGy) for different bacteria irradiated with 1.0 kGy under different modified atmospheric packaging conditions.

	Vacuum-Packed	Nitrogen-Packed	Oxygen-Packed	Air-Packed
*Salmonella* Typhimurium	0.44 ± 0.06	0.38 ± 0.05	0.34 ± 0.04	0.36 ± 0.01
*Escherichia coli*	0.46 ± 0.05	0.40 ± 0.04	0.36 ± 0.05	0.38 ± 0.005

Adapted from Karagöz et al. [51].

**Table 9:** DRD values (in kGy) and ionization dose that can prevent the growth (in kGy) of various yeasts.

Yeasts	DRD (kGy)	Dose ^1^ (kGy)	Treatment Media
*Candida krusei*	-	5.5	Buffer
*Candida spp*	1.25	-	Nutrient broth
*Candida tropicales*	-	10	Buffer
*Candida zelyanoides*	0.7	-	Chicken skin
*Cryptococcus albidus*	-	10	Grape juice
*Debaryomyces klöeckeri*	-	7.5	Grape juice
*Rhodotorula glutinis*	-	10	Grape juice

Adapted from Desmonts et al. [49], ^1^ Dose that can prevent growth (initial number 10^6^–10^7^ cells/mL).

**Table 10:** DRD values (in kGy) and ionization dose that can prevent the growth (in kGy) of various molds.

Molds	DRD (kGy)	Treatment Media	Dose ^1^ (kGy)	Treatment Media
*Alternaria spp*			6	Nutrient agar
*Alternaria citri*	1.2	Lemon		
*Aspergillus flavus*	-	-	5	Peanuts
*A. flavus (spores)*	0.4	Water	1.7	Nutrient broth
*A. flavus*	-	-	6	Sesam seed
*Aspergillus candidus*	0.3	-	-	-
*Aspergillus niger*	0.45	Water	2.5	Nutrient agar
*Botrytis cinerea*	0.6	Plum	5	Nutrient agar
*Cladosporium herbarum*	1	Plum	6	Nutrient agar
*Geotrichum candidum*	0.4	Lemon	-	-
*Penicillium camenbertii*	0.2	Water	-	-
*Penicillium expansum*	0.4	Plum	-	-
*Penicillium roquefortii*	0.4	-	-	-
*Penicillium viridicatum*	-	-	1.4	Nutrient agar
*Rhizopus nigricans*	0.7	Plum	2.5	Nutrient agar

Adapted from Desmonts et al. [49]; ^1^ Dose that can prevent growth (initial number 10^6^–10^7^ cells/mL).

**Table 11:** DRD values (in kGy) for various viruses.

Viruses	DRD (kGy)	Treatment Media	Temperature	References
FMD ^1^ virus types A and C	6	Calf kidney	NI	[49]
FMD virus type D	5	Calf kidney	NI	[49]
FMD virus type O	6	Calf kidney	−60 °C	[49]
FMD virus (A, C, O)	2.7	Buffer	<0 °C	[82]
TESHEN disease virus	4.3	20% peptone solution	NI	[49]
Rinderpest virus	<6	NI ^2^	−20 °C	[49]
Swine fever virus	<6	NI	−20 °C	[49]
Hepatitis A virus	2.72	Lettuce	+4 °C	[81]
Hepatitis A virus	2.92	Strawberry	+4 °C	[81]
Hepatitis A virus	4.83	Oysters	0 °C	[8]
Hepatitis A virus	5.74	Oyster homogenate	0 °C	[8]
Human norovirus	4.05	Oysters	0 °C	[8]
Human norovirus	4.97	Oyster homogenate	0 °C	[8]
Human norovirus	2.55	PBS ^3^	0 °C	[8]
Adenovirus	4.2–5	MEM ^4^ + FBS ^5^	0 °C	[86]
Coxsackievirus	4.2–5	MEM ^4^ + FBS ^5^	0 °C	[86]
Echovirus	4.3–5.5	MEM + FBS	0 °C	[86]
Poliovirus	4.3–5.2	MEM ^4^ + FBS ^5^	0 °C	[86]
Herpes simplex virus	2.92 (β)	Buffer	<0 °C	[82]
Herpes simplex virus	1.1–1.3 (γ)	Buffer	<0 °C	[82]
Rauscher Leukemia Virus	0.6–2.1	Buffer	<0 °C	[82]
Influenza A virus	4.7	MEM ^4^ + FBS ^5^	0 °C	[86]
Rabies virus	1	10% brain emulsion	NI	[49]
Vesicular stomatitis virus	2.7	DMEM ^6^	−78 °C	[50]
La Crosse virus	2.6	DMEM	−78 °C	[50]
Measles virus	2.5	DMEM	−78 °C	[50]
Measles virus	2.7	DMEM	−78 °C	[50]

^1^ Foot and Mouth Disease; ^2^ Not Indicated; ^3^ Phosphate buffered saline, ^4^ Minimum Essential Medium; ^5^ Fetal Bovine Serum; ^6^ Dulbecco’s Modified Eagle Medium, NI not indicated.

**Table 12:** Sterility dose (in kGy) for various parasites.

Parasite	Foods	Parasitic Form	Sterility Dose
*Ascoris lumbricoïdes*	Raw vegetables	Egg	1 to 48
*Anisakis marina*	Mackerel	Larvae	3 to 6
*Cysticercus bovis*	Beef	Larvae	0.2 to 0.6
*Cysticercus cellulosae*	Pork	Destruction	2.8 to 10
*Fasciola hepatica*	Water, vegetables	Cysts forms	0.03
*Fasciola hepatica*		Larvae	1.9
*Fasciola hepatica*		Egg	4.8
*Hymenolepsis nana*	Cereals	Larvae	0.3 to 0.4
*Trichinella spiralis*	Pork	Larvae	0.03 to 0.09
*Trichinella spiralis*		Destruction	1.4 to 6.3

Adapted from Demonts et al. [49], Franssen et al. [90] and Hallman [91].

**Table 13:** Sterilization doses (kGy) for various pests.

Insects Species	Sterilization Dose for Insects		References
	Male (kGy)	Female (kGy)	
*Attagenus unicolor* (Brahm)	0.175	0.175	
*Callosobruchus maculatus*	0.07	0.07	
*Lasioderma serricorne*	0.25	0.175	
*Latheticus oryzae*	0.2	0.1	
*Sitophilus granarius*	0.1	0.1	
*Sitrotoga cereallela*	0.45	0.45	
*Tenebrio molitor*	0.15	0.05	
*Tribolium castaneum*	0.2	0.2	
*Trogoderma glabrum*	0.25	0.132	

Adapted from Demonts et al. [49] and Hallman [91].

**Table 14:** Use of different levels of irradiation dose for food preservation.

Dose (kGy)	Usage for Food Products
0.05–0.15	Anti-germination for potatoes, onions, garlic, shallots, etc.
0.15–3	Pesticide and insecticide properties (by the destruction of embryos and larvae, and sterilization of adults) for cereals, fruits, and pulses. In this dose range, some meat parasitic worms, e.g., *Trichinella* in pork, can be eliminated, and also the ripening of fresh fruits and vegetables is delayed.
2–10	Treatment of meat and meat products, ready meals, fish products (fish, shellfish, frog’s legs), fresh fruit and vegetables, spices and herbs, and various ingredients (gums, additives) to improve and guarantee a hygienic quality by eliminating pathogenic microorganisms and/or extend their shelf life by reducing the microbial population of spoilage.
10–60	Food is sterilized and then can be stored for up to 2 years at room temperature in sealed plastic packaging

**Table 15:** Ionization dose (in kGy) to ensure the radappertization of various foods.

Foods	Dose (kGy)	Temperature
Bacon	25.2	Room
Beef	41.2	−30 °C
Corned beef	26.9	−30 °C
Duck with orange sauce	21	−40 °C
Turkey	28	−40 °C
Ham	31.4	−30 °C
Pork	43.7	−30 °C
Pork sausages	25.5	−30 °C
Chicken	42.7	−30 °C
Mashed carrots and sweet potatoes	28	−40 °C
Madras rice	28	−40 °C

Adapted from Demonts et al. [49].
